# Worldwide Increase of Obesity Is Related to the Reduced Opportunity for Natural Selection

**DOI:** 10.1371/journal.pone.0170098

**Published:** 2017-01-20

**Authors:** Alicja Budnik, Maciej Henneberg

**Affiliations:** 1 Institute of Anthropology, A. Mickiewicz University, Poznań, and Department of Human Biology, Cardinal Stefan Wyszyński University in Warsaw, Poland; 2 Biological Anthropology and Comparative Anatomy Unit, The University of Adelaide, Adelaide, Australia and the Institute for Evolutionary Medicine, University of Zurich, Zurich, Switzerland; University of Missouri Columbia, UNITED STATES

## Abstract

Worldwide rise of obesity may be partly related to the relaxation of natural selection in the last few generations. Accumulation of mutations affecting metabolism towards excessive fat deposition is suggested to be a result of less purging selection. Using the WHO and UN data for 159 countries there is a significant correlation (r = 0.60, p<0.01) between an index of the relaxed opportunity for selection (Biological State Index) and prevalence of obesity (percentage of individuals with BMI >30kg/m^2^). This correlation remains significant (r = 0.32., p<0.01) when caloric intake and insufficient physical activity prevalence are kept statistically constant (partial correlation analysis, N = 82). The correlation is still significant when gross domestic product per capita is also kept constant (r = 0.24, p <0.05, N = 81). In the last decades, prevalence of both obesity and underweight has increased in some countries despite no change in caloric intake nor in physical inactivity prevalence. Relaxed selection against genes affecting energy balance and metabolism may contribute to the increase of fatness independent from commonly considered positive energy balance. Diagnoses of individual predispositions to obesity at an early age and individual counselling on diet and behaviour may be appropriate strategies to limit further increases in body mass.

## Introduction

Obesity prevalence has been increasing though the last several decades worldwide while its causes are not precisely known [1]. This increase is most likely a result of complex interactions between genetic predispositions, environmental factors and human behaviour [2]. Although the main cause of obesity is the disturbed energetic balance—too much food consumed in relation to low field metabolic rates of everyday existence [3]–there are increasingly noticeable differences in adiposity related to individual biological variation. People with larger gastrointestinal tracts accumulate more subcutaneous fat [4,5]. Healthy young adult males whose alanine transaminase activity is elevated have greater BMI values [6,7], there is increase in diabetes among young people [8,9] and the increase in diabetes prevalence remains significant after adjustment for BMI, age and ethnicity [10]. Prevalence of type 1 diabetes correlates with the relaxation of the opportunity for natural selection [11].

Gene-environment interactions play a significant role in regulation of adiposity. Phenotypic variation of body mass contains a substantial genetic component [12,13,14,15]. Recently, a number of genes upregulating metabolism towards excessive fat accumulation have been identified [16,17]. This genetic background to the regulation of body mass underlies hypotheses explaining evolutionary origins of current obesity problem: the ‘thrifty gene’ hypothesis [18,19] and the ‘drifty gene’ hypothesis [20]. The ‘thrifty gene’ hypothesis states that due to periodic food shortages and famines in the past, natural selection increased frequency of genes improving ability to store excess energy into fat, while the ‘drifty gene’ hypothesis states that such genes accumulated when selective pressures for predation avoidance were relaxed in hominin evolution in response to technological developments and improvements in social relations. Both of these hypotheses consider significant time depth for the accumulation of genetic backgrounds to obesity, though the mechanisms they propose can be still active. Another hypothesis has been advanced [12]. It states that humans living in different geographic areas, subjected to different climatic conditions, underwent adaptations to those conditions that are related to energy balance and metabolism. As members of geographic populations migrated recently to other areas of the world, some of their climatic adaptations became disadvantageous in new environments producing obesity.

Here we propose a hypothesis explaining raise of obesity by recent changes in the operation of natural selection. During the last century, the opportunity for natural selection through differential fertility and mortality has been decreasing very substantially [21,22] while it has been found that *de novo* mutations occur at greater rate than previously thought [23] and the mutation load is substantial [24,25]. Purifying selection plays a role in controlling the mutation load [24,25]. lt may be hypothesised that with the decline in the opportunity for selection there is an increasing quantity of heritable factors altering energy balance and metabolism. These may contribute to increasing numbers of obese individuals, as well as some increase in numbers of too lean individuals, even in situations when environmental factors do not promote increasing adiposity of all members of a population The aim of this paper is to investigate a possible coincidence of the relaxation of natural selection and prevalence of obesity.

## Materials and Methods

We have used data from United Nations files (fertility and Gross Domestic Product per Capita) and from World Health Organisation (life tables, prevalence of adults with BMI>30 kg/m^2^, energy [caloric] consumption per capita, prevalence of insufficient physical activity [“physical inactivity” for short], changes in obesity and underweight prevalence through time) for all nations for whom these data were available [26,27] (http://who.int/research/en, http://www.who.int/gho/en/, http://unstats.un.org/unsd/snaama/, http://faostat.fao.org/). A spreadsheet of data used is available as S1 Table.

Biological State Index (I_bs_), [20,21,28] was used as an index of the relaxed opportunity for natural selection in a given national population. Computation of this index requires age specific fertility rates and age-specific survivorship from life tables. Details of the calculation of this index are described in [28,29,30,31]. The index is calculated by combining age-specific death frequency (d_x_ variable of a life table) with an age-specific reproductive loss (s_x_): I_bs_ = 1 − Σd_x_s_x_. Age-specific reproductive loss results from a death of a person at an age before the end of reproductive life span. It is calculated by accumulating to a given age (x) annual age-specific fertility rates, expressing them as a fraction of the Total Fertility Rate and subtracting the result from unity [28,29]: s_x_ = 1- [(Σf_x_)/TFR], where Σf_x_ is the sum of annual age-specific fertility rates up to age “x”, TFR is the total fertility rate, the number of children born to a woman who has reached menopause. When five-year interval age specific fertility rates are used instead of annual ones, their sum is multiplied by five.

Values of s_x_ for sub-adults are by definition equal to 1 because they could not have produced any offspring, for post-reproductive age people s_x_ value is by definition zero. For ages 15–49 years s_x_ values are fractions. For instance, an s_x_ value for a person aged 30 years is the fraction of her fertility that would be lost had she died at that age. For instance, if a woman aged 30 years has already given 3 births, while her total number of births at age 50 is expected to be 6, her s_30_ equals 0.50. Depending on age specific fertility distribution in a given population this s_30_ value may vary from 0.2 (where women limit their fertility after 30) to 0.7 (where women have children later in their lives). Value of 1-s_x_ gives a probability that a person will have an opportunity to pass her/his genes to the next generation. People dying young have lesser opportunity to pass their genes to the next generation, thus lesser reproductive potential, than people surviving to the old age.

The Biological State Index, combining information about mortality by age and reproductive potential by age, gives the probability that an average individual born into a population is able to fully participate in the reproduction of the next generation—to pass her/his genes to the next generation. The lower this probability, the greater the opportunity for natural selection, since the variance in Darwinian fitness (w) is a ratio of individuals who are reproductively unsuccessful to those who are successful [32]. Based on this rule, James Crow introduced in 1958 an index of the opportunity for natural selection through differential mortality, I_m_ [32]. In Crow’s notation I_m_ = Pd/Ps where Pd is the proportion of individuals dying before reaching reproductive age, Ps the proportion of individuals surviving to reproductive age. In terms of I_bs_, which takes into account partial reproductive success during the adult life span, the proportion of reproductively unsuccessful individuals is 1-I_bs_ while proportion of reproductively successful individuals is I_bs_. This is an improvement over the Crow’s index because I_bs_ takes into account the portion of adult mortality that truncates reproductive performance, rather than just assuming that all adults survive through the whole reproductive period. Values of I_bs_ close to 1.00 indicate a loss of opportunity for natural selection through differential mortality because 1-I_bs_ is close to zero. Thus the index is a convenient measure of the relaxation of natural selection—the higher the I_bs_ value, the less opportunity for selection there is.

SPSS version 23.0 was used for statistical analyses.

## Results

**Fig 1:**
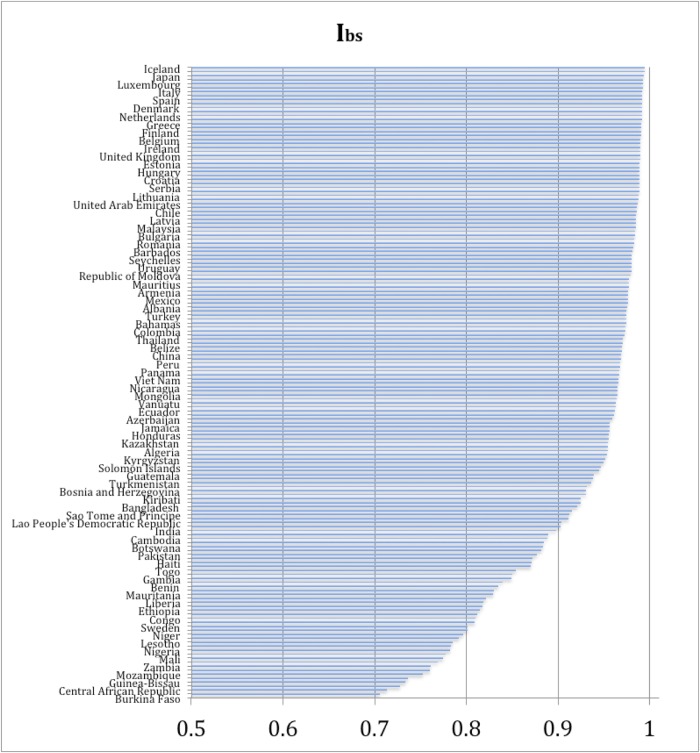
Biological State Index values for 159 countries of the world.

I_bs_ values for individual countries indicate very significant reduction of the opportunity for natural selection in the 21^st^ century (Fig 1). The range of I_bs_ values for this century is from 0.635 (Burkina Faso) to 0.994 (Iceland and Cyprus), its arithmetic mean is 0.927 (sd = 0.080). In 40 (25%) of the countries of the world values of I_bs_ equal at least 0.985, in the next 23 countries (14%) they exceed 0.975. When these values are expressed as the variance of Darwinian fitness (w = [1-I_bs_]/I_bs_) we obtain an average of 0.088. This is four times lower than 100 years ago (0.22, [21]).

**Fig 2:**
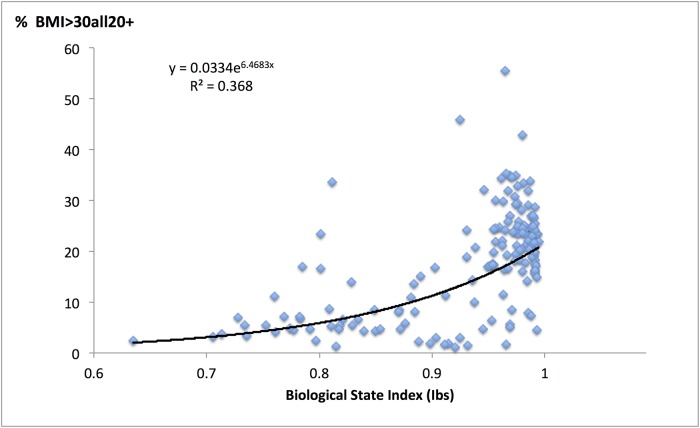
A relationship between the prevalence of obesity and the Biological State Index values.

Regression of obesity prevalence by country on I_bs_ values per country is an exponential function with the correlation coefficient 0.61 (Fig 2, p<0.001). The relationship of similar strength is indicated by the non-parametric Spearman “rho” of 0.56 (p<0.001).

It is obvious that one can expect greater values of I_bs_ and greater prevalence of obesity in more affluent countries, simply because their health services are better and they have greater availability of energy-rich foods. This may cause a spurious correlation of I_bs_ and obesity prevalence. Gross national product per capita, caloric intake and physical inactivity all correlate significantly with the prevalence of BMI>30kg/m^2^ (Table 1). Since Pearson moment-product correlation coefficients for those relationships and for the I_bs_ and obesity are similar to the non-parametric Spearman “rho” coefficients (Table 1), it is possible to use Pearson coefficients to calculate partial correlation between I_bs_ and obesity when the three “confounding” variables are kept statistically constant in various combinations (Table 2). The partial correlation (r = 0.32) between I_bs_ and obesity prevalence remains clearly significant (p<0.01) when both caloric intake and physical inactivity levels are kept constant. The situation is the same when caloric intake and GDP are stabilised (r = 0.24, P<0.01) and even when all three confounders are kept statistically constant, the correlation of the index of selection and obesity prevalence is of similar magnitude (r = 0.24) and remains significant, though at a lower level (p<0.05) due to a smaller sample size caused by missing data on physical inactivity.

**Table 1:** Correlations between obesity (BMI>30 kg/m^2^) prevalence, opportunity for natural selection (I_bs_), GDP per capita, caloric intake and physical inactivity.

Variable:	Obesity	GDP per capita	Nat.selection (I_bs_)	Calories/capita	Physical inactivity
Obesity	1.000	0.654***	0.599***	0.624***	0.403***
GDP per capita	0.614***	1.000	0.688***	0.788***	0.488***
Nat.selection (I_bs_)	0.555***	0.848***	1.000	0.618***	0.382***
Calories per capita	0.591***	0.792***	0.770***	1.000	0.382***
Physical inactivity	0.457***	0.457***	0.309**	0.330**	1.000

*** -significant at p<0.001 level,

**- significant at p<0.01

N = 159 countries, except for physical inactivity where N = 86 countries. All variables were logarithmed to improve homoscedasticity of their distributions. Above diagonal Pearson moment-product correlation coefficients, below the diagonal, Spearman “rho” coefficients.

**Table 2:** Partial correlation between obesity (BMI>30 kg/m^2^) prevalence, and the decreasing opportunity for natural selection (I_bs_), when GDP per capita, caloric intake per capita and physical inactivity are kept statistically constant. All variables were logarithmed to improve homoscedasticity of their distributions.

	Partial correlations of obesity with opportunity for selection	
Variables kept constant	r	N
Calories/capita and physical inactivity	0.316**	82
Calories/capita and GDP	0.242**	155
Calories/capita, GDP and physical inactivity	0.235*	81

** -significant at p<0.01 level,

*—significant at p<0.05 level.

Stepwise multiple regression analysis (SPSS Statistica 23, probability of F to enter < = 0.05, to remove > = 0.10) using obesity prevalence as a dependent variable and gross domestic product, caloric intake, physical inactivity prevalence and I_bs_ as independent variables, selected GDP as the variable having the greatest influence on obesity with R^2^ = 0.421, while opportunity for natural selection (I_bs_) was placed second increasing R^2^ to 0.457. The other variables (caloric intake and physical inactivity) were removed by the analysis as having no statistically significant influence on prevalence of obesity.

## Discussion

Official statistics used by international bodies can only be as accurate as information collected by various national agencies that report them. Thus, due to some technical errors of reporting, ideal correlations can not be expected. The presence of significant correlations in a large sample of countries studied here is indicative of possible interrelationships of variables studied even if values of correlation coefficients are not high.

**Fig 3:**
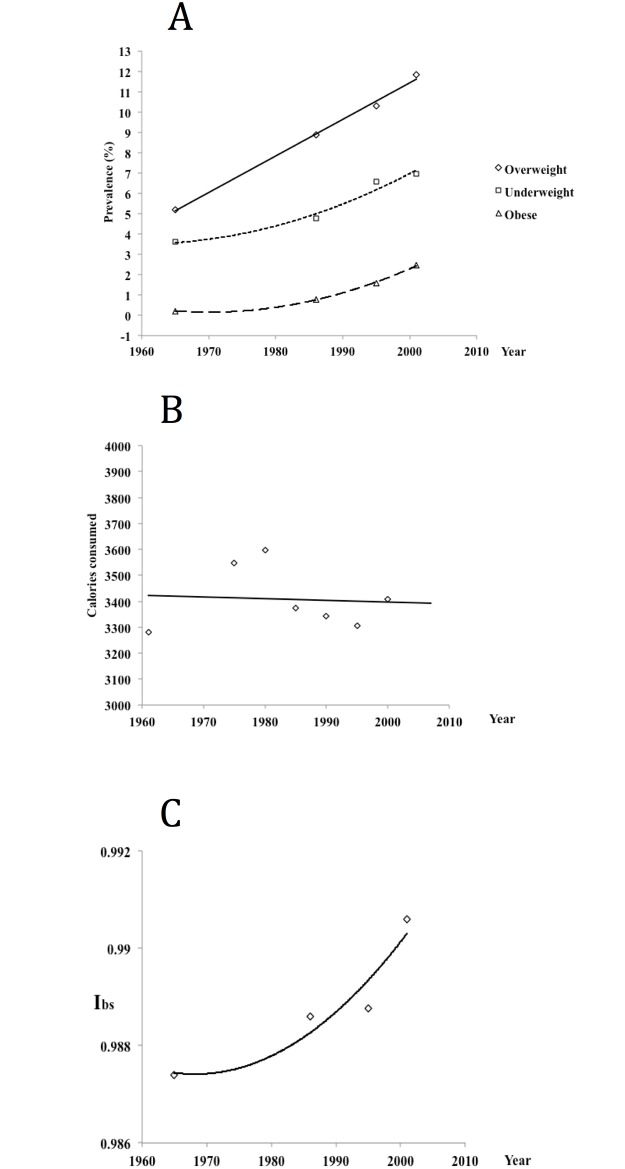
A—changes in the prevalence of overweight (BMI 25.0–29.9 kg/m^2^) obesity (BMI> 30 kg/m^2^) and underweight (BMI < 18.5 kg/m^2^) among Polish conscripts (19 year old males) from 1965 to 2001, B—the average caloric intake in Poland during the same period, C—values of the Biological State Index (I_bs_) in Poland in the same period. All data from [33] except for own calculations of the I_bs_ values.

Parallel changes in the prevalence of obesity and the increase of I_bs_ values during the 20^th^ century in two countries for which data allowing such observations were available, seem to support our observations. In Australia, adult female BMI averages increased from 23 kg/m^2^ in 1926 to 28 kg/m^2^ in 2002 while the I_bs_ values rose in the same period from 0.86 to 0.99 (Table 3). In the analysis of Polish conscript body mass [33] it has been found that prevalence of obesity, overweight and also underweight, has increased from 1965 to 2001 while the caloric intake decreased slightly [33]. In the same period, however, I_bs_ values for the Polish population increased (own calculation from WHO data (Fig 3).

**Table 3:** Average BMI among adult Australian women in 1926, 1995 and in 2002 [34] compared to the values of the biological state index for Australia [21].

Date*	BMI	Ibs
1926	23	0.86
1995	26	0.98
2002	28	0.99

*approximate.

**Fig 4:**
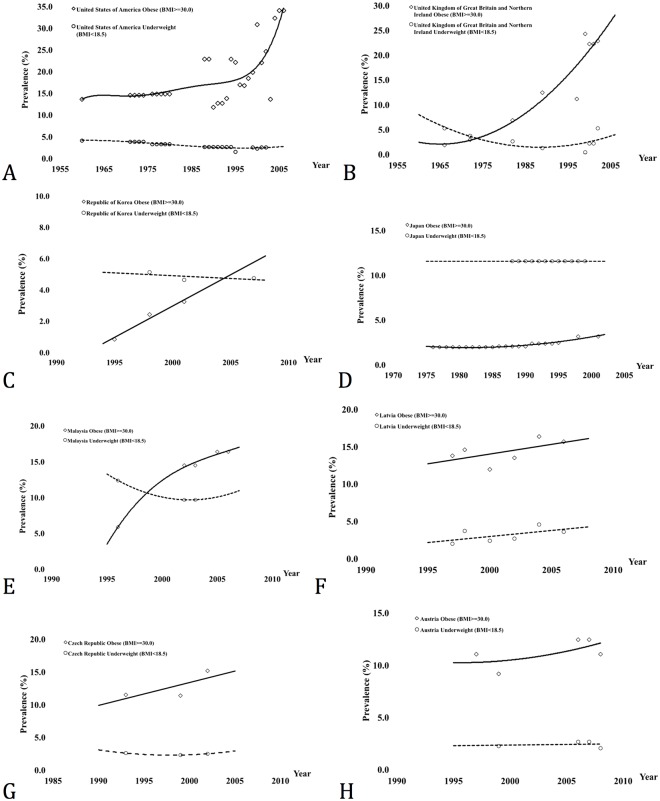
Changes in the prevalence of obesity and underweight in a number of countries. Scales are diferent for each country to reflect range of years and levels of prevalence as given in WHO Global Database on Body Mass Index (BMI): apps.who.int/bmi/index.jsp. Note increases in obesity and increases, or no change, in underweight prevalence. A—United States of America, B—United Kingdom of Great Britain and Northern Ireland, C—Republic of Korea, D—Japan, E- Malaysia, F—Latvia, G—Czech Republic, H—Austria.

In a number of countries (examples in Fig 4) increase in the prevalence of obesity, similar to the Polish situation, has been accompanied by the prevalence of underweight remaining constant or even increasing. With the constant percentage of cases in one extreme category, increase in the frequency of the other extreme category must be a result of the increase in total variance, not just the shift upwards of all values. In everyday terms it is not that everybody is getting fatter, but that the number of fat people increases while many people remain thin. This supports our hypothesis that the number of metabolic faults is increasing. Since the low body mass is now considered a desired feature, increasing numbers of thin people are not as noticeable, or alarming, as those of obese individuals, while some of them may also be a result of metabolic faults.

It is noteworthy that the increases in the prevalence of obesity, and sometimes underweight, may not be paralleled by increased caloric intake nor raising levels of physical inactivity, as suggested by our correlation analysis, though this requires further studies.

Finding of the significant correlation between the values of the Biological State Index and the prevalence of obesity, in the statistically controlled absence of changes in caloric intake and physical inactivity, despite possible inaccuracies of official statistics, indicates a possibility of increasing prevalence of genetically determined metabolic disorders as a consequence of relaxed natural selection with increasing affluence of national populations. Metabolic disorders with genetic background may produce, as one of their signs, increased adiposity. In this situation, public health campaigns aimed at reducing food consumption and increasing exercise, can not be fully effective in reducing prevalence of obesity. It is necessary to improve the understanding of changing metabolic causes of abnormal fat accumulation and to develop effective methods of treatment of increasing adiposity.

## Supporting Information

S1 Table Data on BMI, Ibs, GDP caloric consumption and physical inactivity for individual countries. (XLSX)

## References

[1] Ng M, Fleming T, Robinson M, Thomson B, Graetz N, Margono C, et al Global, regional, and national prevalence of overweight and obesity in children and adults during 1980–2013: a systematic analysis for the Global Burden of Disease Study 2013. The Lancet. 2014 9 5;384(9945):766–81. 10.1016/S0140-6736(14)60460-8 PMC4624264 24880830

[2] Nguyen DM, El-Serag HB. The epidemiology of obesity. Gastroenterology Clinics of North America. 2010; 39(1):1–7. 10.1016/j.gtc.2009.12.014 20202574 PMC2833287

[3] Church TS, Thomas DM, Tudor-Locke C, Katzmarzyk PT, Earnest CP, Rodarte RQ, et al Trends over 5 decades in US occupation-related physical activity and their associations with obesity. PloS one. 2011;6(5):e19657 10.1371/journal.pone.0019657 21647427 PMC3102055

[4] Henneberg M, Ulijaszek SJ, Body frame dimensions are related to obesity and fatness: lean trunk size, skinfolds and body mass index, Am J Hum Biol 2010; 22: 83–91. 10.1002/ajhb.20957 19533618

[5] Lucas T, Henneberg M. Body frame variation and adiposity in development, a mixed longitudinal study of “cape coloured” children. Am J Hum Biol 2014;26: 151–55. 10.1002/ajhb.22494 24307425

[6] Henneberg M, Ruehli FJ, Gruber P, Woitek U. Alanine transaminase individual variation is a better marker than socio-cultural factors for body mass increase in healthy males. Food and Nutrition Sciences 2011; 2 (10): 1054–62.

[7] Grantham JP, KStaub K, Rühli FJ, Henneberg M. Modern Diet and Metabolic Variance—A Recipe for Disaster? Nutrition Journal 2014; 13,15 http://www.nutritionj.com/content/13/1/15 10.1186/1475-2891-13-15 24502225 PMC3923254

[8] Cheng YJ, Imperatore G, Geiss LS, Wang J, Saydah SH, Cowie CC, et al Secular Changes in the Age-Specific Prevalence of Diabetes Among U.S. Adults: 1988–2010 Diabetes Care 2013;36(9): 2690–96. 10.2337/dc12-2074 23637354 PMC3747941

[9] Pettitt DJ, JTalton J, Dabelea D, Divers J, Imperatore G, Lawrence JM, et al Prevalence of Diabetes in U.S. Youth in 2009: The SEARCH for Diabetes in Youth Study. Diabetes Care 2014;37(2): 402–8. 10.2337/dc13-1838 24041677 PMC3898760

[10] Menke A, Rust KF, Fradkin J, Cheng YJ, Cowie CC. Associations Between Trends in Race/Ethnicity, Aging, and Body Mass Index With Diabetes Prevalence in the United States: A Series of Cross-sectional Studies. Ann Intern Med. 2014;161: 328–35. 10.7326/M14-0286 25178569

[11] You WP, Henneberg M. Type 1 diabetes prevalence increasing globally and regionally: the role of natural selection and life expectancy at birth. BMJ Open Diabetes Research & Care. 2016;4(1):e000161. 10.1136/bmjdrc-2015-000161 PMC4780042 26977306

[12] Sellayah D, Cagampang FR, Cox RD. On the evolutionary origins of obesity: a new hypothesis. Endocrinology. 2014;155(5):1573–88. 10.1210/en.2013-2103 24605831

[13] Aguilera CM, Olza J, Gil A. Genetic susceptibility to obesity and metabolic syndrome in childhood. Nutr Hosp. 2013;28(suppl 5):44–55. 24010743 10.3305/nh.2013.28.sup5.6917

[14] Nan C, Guo B, Warner C, Fowler T, Barrett T, Boomsma D, et al Heritability of body mass index inpre-adolescence, young adulthood and late adulthood. Eur J Epidemiol. 2012;27:247–253. 10.1007/s10654-012-9678-6 22426805

[15] Naukkarinen J, Rissanen A, Kaprio J, Pietiläinen KH. Causes and consequences of obesity: the contribution of recent twin studies. International Journal of Obesity. 2012 8 1;36(8):1017–24. 10.1038/ijo.2011.192 21986704

[16] Kunej T, Skok DJ, Zorc M, Ogrinc A, Michal JJ, Kovac M, Jiang Z. Obesity Gene Atlas in Mammals, Journal of Genomics 2012;(1): 45–55. 10.7150/jgen.3996 PMC4091431 25031655

[17] Frayling TM, Timpson NJ, Weedon MN, Zeggini E, Freathy RM, Lindgren CM, et al A common variant in the FTO gene is associated with body mass index and predisposes to childhood and adult obesity. Science 2007 5 11;316(5826): 889–94. 10.1126/science.1141634 17434869 PMC2646098

[18] Neel JV. The “Thrifty Genotype” in 1998l. Nutrition reviews. 1999 5 1;57(5). 10.1111/j.1753-4887.1999.tb01782.x 10391020

[19] Prentice AM, Hennig BJ, Fulford AJ. Evolutionary origins of the obesity epidemic: natural selection of thrifty genes or genetic drift following predation release&quest. International journal of obesity. 2008;32(11):1607–1610. 10.1038/ijo.2008.147 18852700

[20] Speakman JR. Thrifty genes for obesity, an attractive but flawed idea, and an alternative perspective: the ‘drifty gene’hypothesis. International journal of obesity. 2008;32(11):1611–1617. 10.1038/ijo.2008.161 18852699

[21] Stephan C, Henneberg M. Medicine may be reducing the human capacity to survive. Med Hypotheses 2001;57: 633–637. 10.1054/mehy.2001.1431 11735325

[22] Saniotis A. Henneberg M. Medicine could be constructing human bodies in the future. Med Hypotheses 2011;77: 560–564. 10.1016/j.mehy.2011.06.031 21783327

[23] Conrad DF, Keebler JE, DePristo MA, Lindsay SJ, Zhang Y, Cassals F, et al Variation in genome-wide mutation rates within and between human families. Nature genetics. 2011 7 1;43(7):712 10.1038/ng.862 21666693 PMC3322360

[24] Crow J.F. The origins, patterns and implications of human spontaneous mutation. Nature Reviews Genetics 2000;1:40–47. 10.1038/35049558 11262873

[25] Henn BM, Botigué LR, Bustamante CD, Clark AG, Gravel S. Estimating the mutation load in human genomes. Nature Reviews Genetics. 2015 6 1;16(6):333–43. 10.1038/nrg3931 25963372 PMC4959039

[26] http://who.int/research/en.

[27] http://unstats.un.org/unsd/snaama/). (http://faostat.fao.org

[28] Henneberg M. Reproductive possibilities and estimations of the biological dynamics of earlier human populations, J Hum Evol 1976;5: 41–8.

[29] Henneberg M. Comments on the studies of natural increase and biological dynamics of earlier human populations. Anthropos (Athens) 1975;2: 31–9.

[30] Henneberg M, Piontek J. Biological state index of human groups, Przeglad Antropologiczny (Anthrop Review) 1975; 41: 191–201.

[31] Piontek J, Henneberg M. Mortality changes in a Polish rural community (1350–1972) and an estimation of their evolutionary significance. Am J Phys Anthrop 1981;54: 129–38. 10.1002/ajpa.1330540116 7015874

[32] Crow JF. Some possibilities for measuring selection intensities in man. Human Biology. 1958 2 1;30(1):1–3. 13513111

[33] Kozieł S, Szklarska A, Bielicki T, Malina RM. Changes in the BMI of Polish conscripts between 1965 and 2001: secular and socio-occupational variation. Int. J.Obesity. 2006;30:1382–88. 10.1038/sj.ijo.0803292 16534524

[34] Henneberg M, Veitch D. National size and shape survey of Australia. Kinanthreport. 2003;16(1):34–9.

